# Ferroptosis emerges as the predominant form of regulated cell death in goat sperm cryopreservation

**DOI:** 10.1186/s40104-025-01158-0

**Published:** 2025-02-18

**Authors:** Erhan Hai, Boyuan Li, Yukun Song, Jian Zhang, Jiaxin Zhang

**Affiliations:** Inner Mongolia Key Laboratory of Sheep & Goat Genetics Breeding and Reproduction, College of Animal Science, Inner Mongolia Agricultural University, Hohhot, Inner Mongolia 010018 China

**Keywords:** Cashmere goat, Ferroptosis, Sperm cryo-damage

## Abstract

**Background:**

Freezing-induced sperm damage, often associated with oxidative stress, can result in regulated cell death. Given that oxidative stress can trigger various forms of regulated cell death, the prevailing form during sperm cryopreservation remains unknown. Our study aimed to investigate this issue using cashmere goats as a model.

**Results:**

We found a significant increase in lyso-phospholipids in frozen-thawed sperm suggested ferroptosis. Assessment of cryopreserved sperm, with or without prior treatment with ferroptosis or apoptosis inhibitors, demonstrated the significant efficacy of ferroptosis inhibitors in reducing freezing damage. This implicates ferroptosis as the primary form of regulated cell death induced during sperm cryopreservation. Additionally, the positive rate of transferrin receptor protein 1 was significantly lower in fresh live sperm (47.8%) than in thawed live sperm (71.5%), and the latter rate was lower than that in dead sperm (82.5%). By contrast, cleaved caspase-3 positivity showed no significant difference between fresh live sperm and thawed live sperm but was notably lower in thawed live sperm than in dead sperm.

**Conclusions:**

Our findings establish ferroptosis as the dominant regulated cell death form during goat sperm cryopreservation, providing novel insights into freezing-induced sperm damage mechanisms. These findings have significant implications for optimizing cryopreservation protocols and enhancing sperm viability after freezing-thawing.

**Graphical Abstract:**

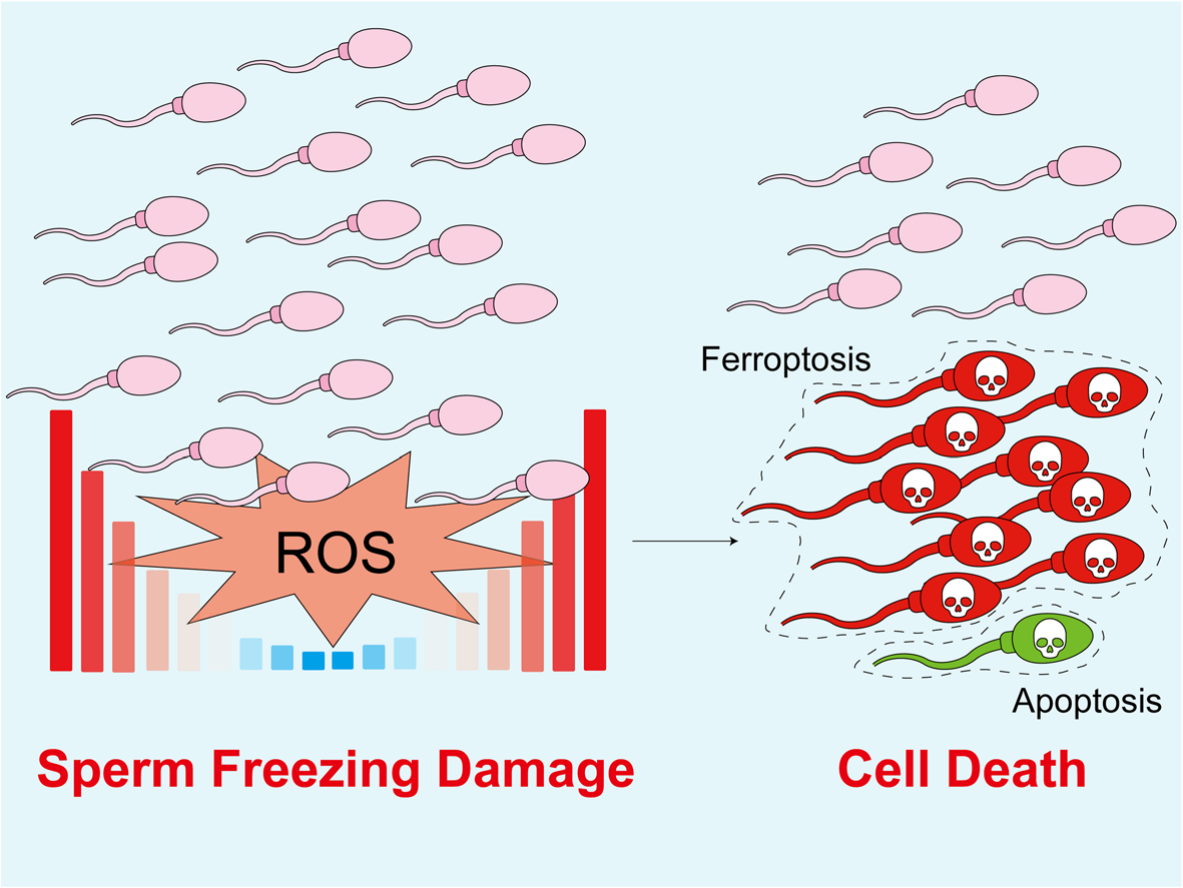

**Supplementary Information:**

The online version contains supplementary material available at 10.1186/s40104-025-01158-0.

## Introduction

Sperm cryopreservation is a vital technique for preserving gamete resources and advancing artificial insemination technology [1, 2].

Despite advances in cryopreservation methods, a substantial proportion of sperm (40%–50%) fail to survive the freezing process; surviving populations often exhibit impaired physiological function post-thaw [3, 4], known as sperm freezing damage. This phenomenon poses a significant hurdle in reproductive biology.

Oxidative stress is a leading cause of sperm freezing damage, which compromises both the structural and functional integrity of sperm. It results from an imbalance regarding intracellular redox reactions [5, 6]. The reactive oxygen species (ROS) generated during normal physiological processes are crucial for cell signaling and tissue homeostasis [7]. In mature sperm, ROS are primarily produced through two pathways: the nicotinamide adenine dinucleotide phosphate oxidase system and electron leakage from the mitochondrial electron transport chain [8–10]. The role of ROS in sperm depends on its concentration; in moderation, it aids in normal physiological functions such as chromatin stability, DNA protection, sperm capacitation, and forward motility [11]. However, excessively high levels of ROS lead to oxidative stress, triggering lipid peroxidation of the sperm plasma membrane and mitochondrial damage, which subsequently causes DNA injury known as "oxidative damage" [6].

The oxidative stress during sperm freezing arises from two main sources [12]: firstly, the depletion of antioxidants. Sperm, highly differentiated cells with compact chromatin, lack a genomic antioxidant response [13]. Secondly, disruption of the extracellular microenvironment. During cryopreservation, the temperature decrease and dehydration reduce the sperm metabolic rate, enhancing survival [12]. Upon thawing, however, the restoration of the extracellular environment and metabolic activity leads to a rapid surge in ROS. Without appropriate control, this surge can cause oxidative stress and culminate in sperm death [1, 10, 14].

Oxidative stress can induce apoptosis, the only confirmed form of regulated cell death (RCD) during sperm cryopreservation [12, 15]. Studies have demonstrated changes in apoptosis-related markers during semen cryopreservation [16]. Apoptosis is regulated by the caspase family and can be categorized as endogenous or exogenous, both leading to cleaved caspase-3 (CL-caspase3) activation [17]. Caspase family inhibitors, such as Z-VAD-FMK (Z-VAD), effectively inhibit apoptosis [18]. However, pre-cryopreservation addition of Z-VAD has no significant effect on plasma membrane integrity in frozen-thawed bovine sperm, and post-thawing addition does not significantly improve sperm viability [19]. Similarly, adding Z-VAD before or after cryopreservation has no effect on sperm viability or plasma membrane integrity in cryopreserved canine sperm [20]. Considering the association of oxidative stress with various forms of RCD, non-apoptotic RCD may occur during sperm cryopreservation [17].

Among the factors contributing to cellular oxidative stress, lipid peroxidation in biological membranes, particularly of polyunsaturated fatty acids (PUFAs), has emerged as a critical regulator of cellular fate [21]. This process can lead to cell death through a recently identified form of RCD known as "ferroptosis" [22]. Ferroptosis is characterized by iron-dependent lipid peroxidation reaching lethal levels, specifically targeting PUFA-phospholipids (PUFA-PLs) in biological membranes [21, 22].

Understanding the role of ferroptosis in sperm freezing damage is crucial as it could offer novel insights into mitigating sperm damage and improving cryopreservation protocols. While the relationship between ferroptosis and sperm freezing damage remains unclear, the high susceptibility of PUFAs in the plasma membrane of mature sperm to oxidation [23–27] suggests that ferroptosis may be a promising avenue for research on sperm freezing damage. Additionally, several ferroptosis inhibitors—such as coenzyme Q10, α-tocopherol (α-TOH), and Trolox—have demonstrated effectiveness in enhancing sperm cryopreservation outcomes [12]. Key ferroptosis regulatory proteins, including glutathione peroxidase 4 (GPX4) and peroxiredoxin 6 (PRDX6), have also been implicated in protecting against sperm freezing damage [28].

This study aims to investigate the role of ferroptosis in sperm freezing damage in Inner Mongolia cashmere goats. We will identify the primary RCD type associated with freezing damage through metabolomics; examine the effects of RCD inhibitors on sperm survival after cryopreservation; and elucidate primary RCD type during cryopreservation by analyzing marker proteins.

## Materials and methods

### Semen collection, cryopreservation, and thawing

**Fig. 1:**
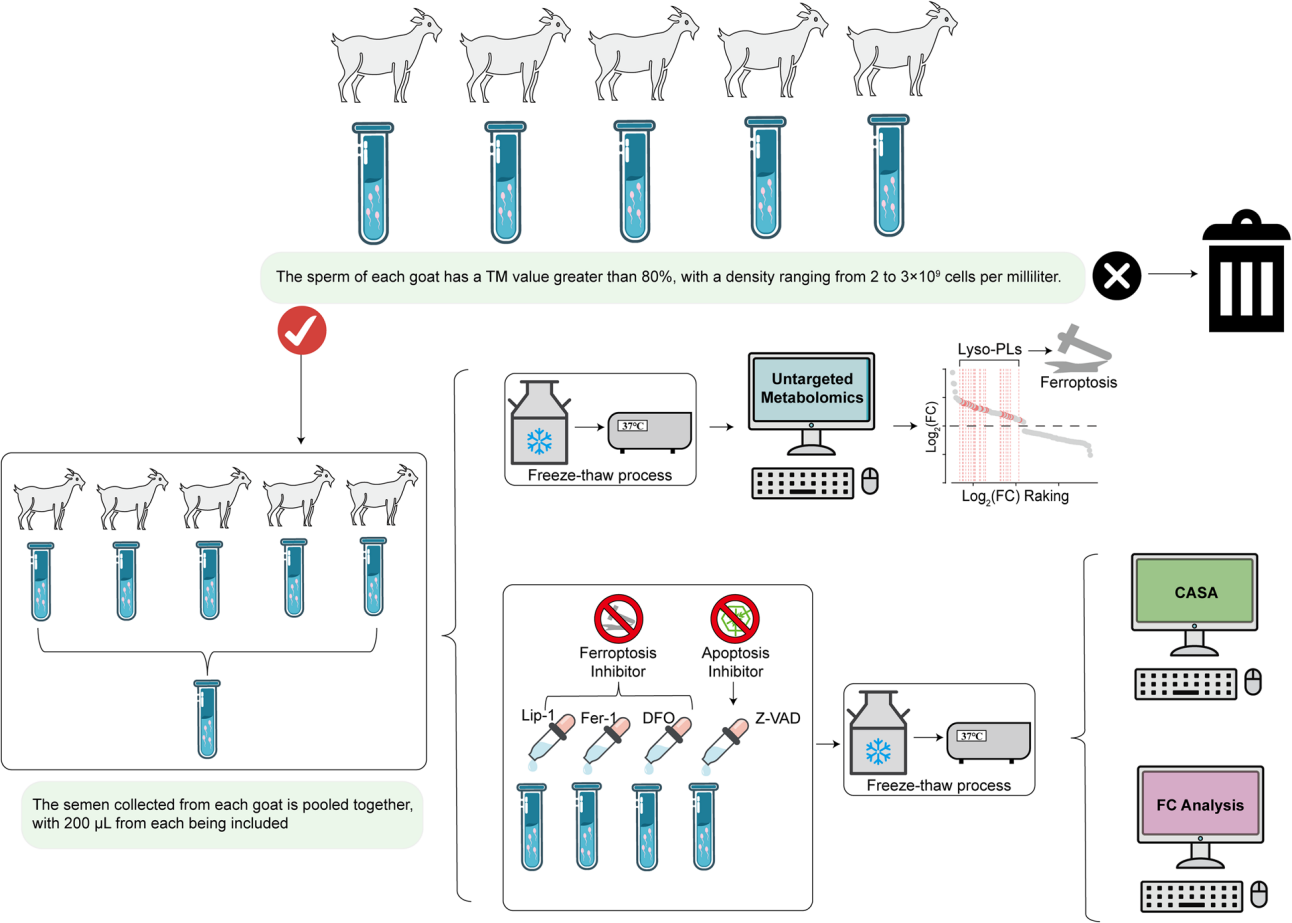
The flowchart for the overall experiment

The flowchart for the overall experiment is shown in Fig. 1. From mid-August to early November 2023, during the breeding season, semen samples were obtained from five healthy 2-year-old Inner Mongolia cashmere goats sourced from Inner Mongolia Jinlai Animal Husbandry Technology Co., Ltd. (Hohhot, China). This period is characterized by moderate temperatures and relatively low precipitation, creating favorable conditions for goat reproductive activity and the collection of high-quality semen samples. Semen collection was conducted three times weekly during this period. The goats were maintained under standard management conditions; feeding occurred twice daily and water was available ad libitum.

On each collection day, goat semen was obtained using the artificial vagina method. Immediately after collection, a comprehensive quality assessment of the fresh semen was conducted using a computer-assisted sperm analysis system (CASA, IVOS II, IMV Technologies, France) to evaluate sperm motility and a sperm density meter (IMV Technologies) to determine sperm concentration. Only semen samples from all five goats that met the strict criteria of total motility exceeding 80% and a sperm density of 2–3 × 10^9^ cells/mL on the same day were used for experiments. Qualified semen samples (200 μL per goat) collected on the same day were pooled in a sterile container to create a single mixed sample, representing one biological replicate.

After collection, semen samples (200 μL per goat) were pooled and maintained at 37 °C. The pooled semen was diluted to a sperm density of 2 × 10^8^ cells/mL using a standard semen diluent. The composition of the diluent and the procedures for cryopreservation and thawing followed previously established protocols [29]. The semen diluent consisted of Tris (1.8 g), citric acid (1 g), glucose (0.5 g), penicillin–streptomycin (0.5 mL; 15140122, Gibco, USA), 6% glycerol (3 mL), egg yolk (15 mL; Charoen Pokphand Group, China), and ultrapure water to a final volume of 50 mL. Unless otherwise specified, reagents were sourced from Sigma-Aldrich (USA). The cryopreservation process began with semen dilution, followed by gradual cooling of the diluted semen to 4 °C within 2 h. After the desired temperature had been reached, the semen was loaded into straws, sealed with sealing powder yellow (018815, IMV Technologies, France), and equilibrated for 1 h. The straws were then placed 4 cm above liquid nitrogen and exposed to nitrogen vapor for 7 min prior to storage in liquid nitrogen. To thaw the frozen semen, the straws were immersed in a 37 °C water bath for 30 s after 7 days of storage.

### Non-targeted metabolomics analysis

Non-targeted metabolomics analysis was conducted to identify differential metabolites between the fresh and frozen-thawed goat sperm samples. Each pooled semen sample, described in the Section *Semen collection, cryopreservation, and thawing* of the methodology, was diluted according to the methods outlined in that section. After 15 min, the sample was divided into two equal portions. One portion formed the fresh semen group (Fresh group), while the other portion underwent the freezing and thawing procedure, becoming the frozen-thawed semen group (frozen-thawed group). Each experimental day constituted a biological replicate, and both groups contained six such replicates. Each group underwent centrifugation at 300 × *g* and 4 °C for 5 min after the addition of phosphate-buffered saline (PBS). This step was repeated 2–3 times to remove the mixture of seminal plasma and diluent. Subsequently, samples were rapidly immersed in liquid nitrogen for 3–5 min before long-term storage at −80 °C. Upon thawing on ice, metabolites were extracted from the samples using a pre-cooled 80% methanol buffer. Extraction mixtures were subsequently stored at −20 °C for 30 min before centrifugation at 20,000 × *g* and 4 °C for 15 min. Supernatants were vacuum-dried, redissolved in 100 μL of 80% methanol, and stored at −80 °C until further analysis. Additionally, 10-μL aliquots of each extract were pooled to prepare a combined quality control (QC) sample mixture.

For metabolite identification, liquid chromatography– mass spectrometry (LC–MS) was employed. Chromatographic separation was conducted using a Vanquish Flex UPLC system (Thermo Fisher Scientific, Bremen, Germany) equipped with an ACQUITY UPLC T3 column (100 mm × 2.1 mm, 1.8 µm, Waters, Milford, MA, USA). High-resolution mass spectrometry was performed using a Q-Exactive high-resolution tandem mass spectrometer (Thermo Fisher Scientific, Bremen, Germany) operating in both positive and negative ion modes. During the acquisition process, a QC sample scan was conducted every 10 samples to ensure data quality. The samples were stored at −80 °C prior to LC–MS analysis. Pooled QC samples were also prepared by combining 10 μL of each extraction mixture.

Raw mass spectrometry data underwent preprocessing using Proteowizard's MSConvert software (v4.5). Peak extraction and QC were performed using XCMS software (v3.5), and the CAMERA (v1.6) package in R (v4.1.0) was utilized for adduct and ion annotation. Metabolite identification was conducted using MetaX software (v1.3.5), matching primary mass spectrometry information to databases for identification and secondary mass spectrometry information to an in-house standard database for verification. Identified substances were annotated using databases such as the Human Metabolome Database (https://hmdb.ca/) and the Kyoto Encyclopedia of Genes and Genomes (https://www.kegg.jp/). MetaX software (v1.3.5) was employed to quantify the metabolites and screen for differential metabolites.

### Addition of ferroptosis and apoptosis inhibitors in diluent

Semen collection, cryopreservation, and thawing procedures were conducted as described in Section *Semen collection, cryopreservation, and thawing*. Inhibitors were added to the diluent before cryopreservation. To investigate the effects of RCD inhibitors on sperm freezing damage, four specific inhibitors were utilized at concentrations of 2 μmol/L, 5 μmol/L, and 10 μmol/L, which were determined based on the manufacturers’ recommendations. The inhibitors included the apoptosis inhibitor Z-VAD (CM00937) and three ferroptosis inhibitors: Liproxstatin-1 (Lip-1, CM04076), Ferrostatin-1 (Fer-1, CM00719), and deferoxamine (DFO, CM00682). All four were sourced from Proteintech (Wuhan, China). Each inhibitor was dissolved in dimethyl sulfoxide (DMSO) and then added to the diluent at the specified final concentration. This approach resulted in 12 experimental groups (4 inhibitors × 3 concentrations), enabling a systematic evaluation of the protective effects of these inhibitors against sperm freezing damage.

Additionally, to ensure the validity of the results, two control groups were established: C1 served as the diluent-only control, whereas C2 was treated with the diluent supplemented with 0.1% DMSO, which was the maximum DMSO concentration present in any of the inhibitor-treated samples.

### Sperm motility assessment

Sperm motility was assessed immediately after thawing. Sperm motility parameters were assessed using a CASA (IVOS II, IMV Technologies), as described previously [29]. Specifically, 3 μL of the frozen-thawed sperm sample were gently placed on a preheated Leja slide chamber and then in an advanced CASA system. Prior to analysis, the CASA system was calibrated according to established parameters and standards to guarantee data accuracy. Definitions for these sperm motility parameters were provided in the work by Dorado et al. [30]; the customized settings used for goat sperm are detailed in Table 1. For each sperm sample, at least 10 random fields of view were selected for assessment, ensuring the sample was adequately represented. Within each field, the system automatically tracked and calculated the motion trajectories and parameters of at least 1,000 sperm. The primary motion parameters measured included total motility (TM, %), progressive motility (PM, %), average path velocity (VAP, μm/s), linear motion velocity (VSL, μm/s), curvilinear motion velocity (VCL, μm/s), straightness (STR, %) and linearity (LIN, %), amplitude of lateral head displacement (ALH, μm), and beat cross frequency (BCF, Hz).

**Table 1:** Setting for goat semen analyses

Features	Setting
Frame capture, Hz	60
Frame count	30
Headsizemax, μm^2^	70
Headsizemin, μm^2^	6
Elongation max, %	100
Elongation min, %	1
Slow VAP, μm/s	20
Slow VSL, μm/s	30
Max photometer	70
Min photometer	60
Progressive STR	80
Progressive VAP, μm/s	30
Static VAP, μm/s	4
Static VSL, μm/s	1
Head brightness, min	200
Tail brigthness, min	96
Maximum width to length ratio, %	90
Minimum width to length ratio, %	1
Temperature, °C	37

Within each inhibitor group, the optimal inhibitor concentration for subsequent studies was determined based on sperm samples exhibiting the highest TM and PM values.

### Flow cytometric assessment of RCD, ROS, lipid peroxidation, and Fe^2+^ levels, as well as plasma membrane and acrosome integrity

A CytoFLEX flow cytometer (Beckman Coulter, Brea, CA, USA), was used to analyze various cellular parameters, including the levels of RCD, ROS, lipid peroxidation, Fe^2+^ concentrations, plasma membrane integrity, and acrosome status in the presence of various inhibitors. The study was divided into six experimental groups: C1, C2, and four groups each with the optimal concentration of a different RCD inhibitor. On each experimental day, the mixed semen samples were split into six equal portions, each of which was assigned to one of the six groups. To ensure statistical robustness, all experiments within each group were repeated at least six times, with each experimental day representing an independent biological replicate.

#### Assessment of plasma membrane permeability

Plasma membrane integrity was evaluated using Fixable Viability Dye eFluor™ 780 (FVD, 65-0865-14, Thermo Fisher Scientific) [31]. For each group, 100 μL of thawed semen was centrifuged at 300 × *g* for 5 min. After the supernatant had been discarded, the pellet was washed with PBS. The sperm concentration was adjusted to 2 × 10^6^ sperm/mL using 499 μL of non-capacitating sperm BWW culture medium (NC-BWW medium, Genemed Biotechnologies, San Francisco, CA, USA) and 1 μL of 10 mmol/L FVD. The mixture was incubated in the dark at 37 °C for 10 min. After incubation, cells were washed as described above and resuspended in 200 μL of NC-BWW medium prior to flow cytometric analysis. NC-BWW medium provides an optimal environment for sperm, minimizing potential cell damage caused by processing [32, 33]. The flow cytometer used a 638-nm excitation light as the source for FVD and used a 780/60 BP filter to collect signal intensity. The sample flow rate was maintained at 200–400 particles/s; at least 10,000 cells were analyzed in each sample.

#### Assessment of acrosomal integrity

Acrosomal integrity was evaluated using a peanut agglutinin detection kit (PNA-FITC, Genemed Biotechnologies) [34]. Thawed semen was incubated at 37 °C for 30 min, then centrifuged at 300 × *g* for 5 min. After the supernatant had been discarded, the sperm concentration was adjusted to 2 × 10^7^ sperm/mL using GENMED preservative solution. Subsequently, a 100-μL aliquot of the suspension was combined with 500 μL of GENMED cleanup solution. After additional centrifugation at 300 × *g* for 5 min, the supernatant was removed, and cells were resuspended in 200 μL of GENMED staining solution B. Samples were incubated in the dark at room temperature for 20 min and centrifuged again. The supernatant was discarded, and 200 μL of propidium iodide (PI, 0.4 μg/mL) was added. Samples were incubated in the dark at room temperature for 5 min and then centrifuged; the supernatant was removed, and 1 mL of GENMED cleanup solution was added. The samples were then subjected to flow cytometric analysis. The flow cytometer used a 488-nm excitation light as the source for both PNA-FITC and PI. A 525/40 BP filter was used to collect the signal intensity of PNA-FITC; a 585/42 BP filter was utilized to collect the signal intensity of PI. The sample flow rate was maintained at 200–400 particles/s; at least 10,000 cells were analyzed in each sample. Single-stained tubes with PNA-FITC and PI were prepared for fluorescence compensation.

#### Annexin V-APC/PI assessment of RCD

An Annexin V-APC/PI kit (88-8007-74, Franklin Lakes, NJ, USA) was used to detect the percentage of cells in each RCD state [35]. For each group, 100 μL of thawed semen was centrifuged at 300 × *g* for 5 min. After the supernatant had been discarded, the pellet was washed with PBS. The sperm concentration was adjusted to 2 × 10^6^ sperm/mL using 495 μL of Annexin V Binding Buffer. To this suspension, 5 μL of APC-Annexin V was added; the sample was thoroughly mixed and incubated in the dark at 37 °C for 10 min. Subsequently, 5 μL of PI Staining Solution was added, and the sample was incubated under the same conditions for an additional 5 min. Cells were washed again as described above, resuspended in 200 μL of NC-BWW medium, and subjected to flow cytometric analysis. The flow cytometer used a 638-nm excitation light as the source for APC-Annexin V and a 488-nm excitation light as the source for PI. A 660/20 BP filter was used to collect the signal intensity of Annexin V, whereas a 690/50 BP filter was utilized to collect the signal intensity of PI. The sample flow rate was maintained at 200–400 particles/s per second; at least 10,000 cells were analyzed in each sample. Single-stained tubes with Annexin V-APC and PI were prepared to perform fluorescence compensation.

#### Assessment of ROS levels

ROS levels in live cells were measured using a ROS kit (S0033S, Beyotime, Shanghai, China) and FVD [36]. For each group, 100 μL of thawed semen was centrifuged at 300 × *g* for 5 min. Supernatants were discarded, and pellets were washed with PBS. The sperm concentration was adjusted to 2 × 10^6^ sperm/mL using 499.5 μL of NC-BWW medium (containing 1 μL of 10 mmol/L FVD) and 0.5 μL of 10 mmol/L 2′,7′-dichlorodihydrofluorescein diacetate (DCFH-DA), and mixtures were incubated in the dark at 37 °C for 10 min. Cells were washed as above and resuspended in 200 μL of NC-BWW medium and subjected to flow cytometric analysis. The flow cytometer used a 638-nm excitation light as the source for FVD and a 488-nm excitation light as the source for DCFH-DA. A 525/40 BP filter was used to collect the signal intensity of DCFH-DA, whereas a 780/60 BP filter was used to collect the signal intensity of FVD. The sample flow rate was maintained at 200–400 particles/s; at least 10,000 cells were analyzed in each sample.

#### Assessment of lipid peroxidation

Lipid peroxidation levels in live cells were measured using the BODIPY™ 581/591 C11 lipid peroxidation sensor (D3861, Thermo Fisher Scientific) and FVD [37]. For each group, 100 μL of thawed semen was centrifuged at 300 × *g* for 5 min. Supernatants were discarded, and pellets were washed with PBS. The sperm concentration was adjusted to 2 × 10^6^ sperm/mL using 499.5 μL of NC-BWW medium (containing 1 μL of 10 mmol/L FVD) and 0.5 μL of BODIPY C11 (10 mmol/L), and mixtures were incubated in the dark at 37 °C for 10 min. Cells were washed as above and resuspended in 200 μL of NC-BWW medium and subjected to flow cytometric analysis. The flow cytometer used a 638-nm excitation light as the source for FVD and a 488-nm excitation light as the source for oxidized C11-BODIPY (oxC11-BODIPY). A 525/40 BP filter was used to collect the signal intensity of oxC11-BODIPY, whereas a 780/60 BP filter was used to collect the signal intensity of FVD. The sample flow rate was maintained at 200–400 particles/s; at least 10,000 cells were analyzed in each sample.

#### Assessment of Fe^2+^

Fe^2+^ levels in live cells were measured using a cellular ferrous ion detection kit (FerrOrange, F374, Dojindo, Kumamoto, Japan) and FVD [38]. For each group, 100 μL of thawed semen was centrifuged at 300 × *g* for 5 min. The supernatants were discarded, and the pellets were washed with PBS. The sperm concentration was adjusted to 2 × 10^6^ sperm/mL using 495 μL of NC-BWW medium (containing 1 μL of 10 mmol/L FVD) and 5 μL of FerrOrange (1 mmol/L), and the mixtures were incubated in the dark at 37 °C for 10 min. Cells were washed as above, resuspended in 200 μL of NC-BWW medium, and subjected to flow cytometric analysis. The flow cytometer used a 638-nm excitation light as the source for FVD and a 488-nm excitation light as the source for Fe^2+^. A 585/42 BP filter was used to collect the signal intensity of Fe^2+^, whereas a 780/60 BP filter was used to collect the signal intensity of FVD. The sample flow rate was maintained at 200–400 particles/s; at least 10,000 cells were analyzed in each sample.

### Flow cytometric analysis of key ferroptosis- and apoptosis-associated proteins

We investigated dynamic changes in the expression of key proteins involved in ferroptosis regulation within live sperm cells. Specifically, we examined transferrin receptor (TFRC), CL-caspase3, GPX4, and ferritin heavy chain (FTH1), comparing their expression levels between fresh sperm and frozen-thawed sperm. Notably, TFRC expression is upregulated in cell models where ferroptosis is induced through various pathways, providing a clear distinction from cells undergoing apoptosis [21, 39]. Thus, TFRC-positive cells can serve as a marker for cells undergoing ferroptosis.

We also analyzed the distribution of these proteins in live and dead sperm populations within the frozen-thawed sperm samples. By examining the expression levels of TFRC, FTH1, CL-caspase3, and GPX4 in these distinct populations, we aimed to clarify the role of these proteins in modulating the primary form of RCD that occurs during sperm freezing damage. GPX4, a key regulator of ferroptosis, exerts its inhibitory effects by reducing glutathione (GSH) [21, 40]; its expression was evaluated in the present study. Additionally, ferroptosis is characterized by ferritin autophagy, a process in which ferritin, an iron-storage protein, is degraded within cells [41, 42]. Finally, FTH1 expression was assessed in frozen-thawed sperm samples.

Flow cytometry was employed to analyze intracellular protein expression in sperm cells, comparing fresh sperm with frozen-thawed sperm. This analysis was further stratified to distinguish between live and dead sperm within the frozen-thawed sperm population, providing insights into how cryopreservation affects protein expression. Flow cytometry intracellular fixation and permeabilization buffers were obtained from Thermo Fisher Scientific (88-8824-00). The primary antibodies included TFRC (66180-1-lg, Proteintech), FTH1 (ab75973, Abcam, Cambridge, UK), CL-caspase3 (9661, Cell Signaling Technology), GPX4 (30388-1-AP, Proteintech), rabbit polyclonal IgG isotype control (30000-0-AP, Proteintech), and mouse monoclonal IgG2α isotype control (61656, Cell Signaling Technology, Danvers, MA, USA). The isotype control for TFRC was mouse monoclonal IgG2α isotype control, whereas the isotype controls for CL-caspase3, FTH1, and GPX4 were rabbit polyclonal IgG isotype controls. The secondary antibodies included CoraLite488-conjugated donkey anti-rabbit IgG (SA00013-6, Proteintech) and donkey anti-mouse IgG (SA00013-5, Proteintech).

Fresh sperm and frozen-thawed sperm were labeled for viability using FVD following the method described in Section *Assessment of plasma membrane permeability*. After labeling, cells were washed with PBS and subjected to fixation and permeabilization steps. Specifically, 200 μL of fixation buffer was added to the cells following incubation in the dark at room temperature for 30 min. Next, 300 μL of permeabilization buffer was added, and samples were centrifuged at 300 × *g* for 5 min. This step was repeated twice, and then 400 μL of permeabilization buffer was added, followed by incubation in the dark at room temperature for 15 min. Following centrifugation, cells were blocked in 100 μL of permeabilization buffer and 400 μL of BSA (10%) in the dark at room temperature for 30 min.

After blocking, washed cells were incubated with primary antibodies (final concentration of 5 μg/mL) in 200 μL of permeabilization buffer for 60 min in the dark at room temperature. Cells were then washed with permeabilization buffer, followed by incubation with secondary antibodies (1:200) in 200 μL of permeabilization buffer for 30 min in the dark at room temperature. Following additional washes, cells were resuspended in 200 μL of NC-BWW medium.

Samples were analyzed using flow cytometry with a rigorous protocol that ensured the detection of 10,000 cells per group. During this process, the expression level of each protein was determined by measuring the intensity above its respective isotype control. All target proteins were assayed via the FITC channel. FlowJo (v10.8.1) software was utilized to visualize and analyze the flow cytometry data. This software facilitated both raw data visualization and the execution of t-distributed stochastic neighbor embedding (tSNE) analysis. The FITC and APC-750 channels were selectively employed for the tSNE analysis using default parameter conditions optimized for general applicability while preserving sensitivity and clarity in the resulting embeddings. Furthermore, GraphPad Prism (v10.1.0) software was leveraged to generate heatmaps, which provided an intuitive representation of the protein expression patterns across samples.

### Statistical analysis

We used the OmicStudio tools (available at https://www.omicstudio.cn/tool) to perform orthogonal partial least squares discriminant analysis (OPLS-DA) of secondary metabolites. After the OPLS-DA model had been established, we conducted 200 iterations of seven-fold cross-validation to assess its robustness and predictive performance. Subsequently, we calculated variable importance in projection (VIP) values for each variable in the model to determine their contributions to the classification. All statistical analyses were performed using SPSS Statistics 29.0 (IBM, Armonk, NY, USA) for Windows. Before conducting parametric tests, we evaluated data normality using the Shapiro–Wilk test and assessed variance homogeneity with the Levene test. To identify quantitative differences in metabolites between fresh semen and frozen-thawed semen, we utilized an unpaired two-tailed *t*-test. For sperm motility comparisons, we used repeated measures and one-way analysis of variance (ANOVA) to assess differences between groups. For other experiments, we utilized ANOVA to compare groups; post hoc analysis with Tukey’s honestly significant difference method was performed to identify specific differences between experimental groups. A significance threshold of *P* < 0.05 was considered statistically significant for all tests. Figures were generated and edited using GraphPad Prism 10 (GraphPad Software, San Diego, CA, USA). The gating strategy for analyzing the results of flow cytometry in this research section is detailed in Additional file 1.

## Results

### Non-targeted metabolomics reveals a link between ferroptosis and sperm freezing damage

**Fig. 2:**
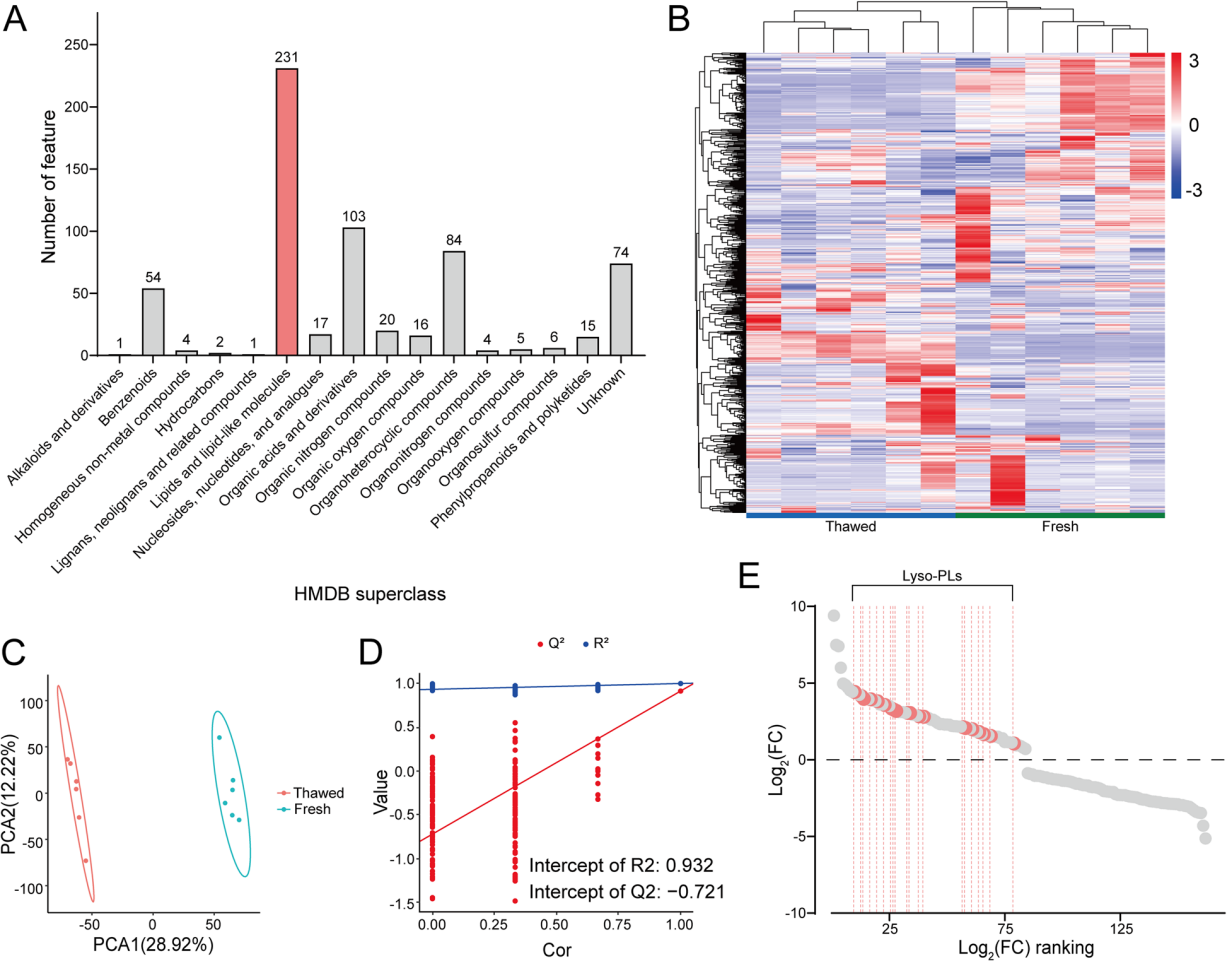
Non-targeted metabolomics analysis of fresh and frozen-thawed goat sperm. **A** Classification of secondary metabolites according to the Human Metabolome Database (HMDB) Super Class. **B** Heatmap depicting the expression profiles of secondary metabolites in fresh and frozen-thawed sperm. **C** Score plot of Partial Least Squares Discriminant Analysis (PLS-DA) differentiating between fresh and frozen-thawed sperm based on metabolomic profiles. **D** Permutation test plot assessing the reliability of the PLS-DA model. **E** Scatter plot ranking significantly different metabolites between fresh and frozen-thawed sperm, based on fold-change values

Non-targeted metabolomics analysis was conducted on fresh and frozen-thawed sperm samples to explore non-apoptotic forms of RCD associated with sperm freezing damage. A total of 637 secondary metabolites were identified (Fig. 2A and B), with lipids and lipid-like molecules comprising the largest category, totaling 231 species.

OPLS-DA highlighted significant differences in the metabolomic profiles of sperm before and after cryopreservation (Fig. 2C), without signs of overfitting (Fig. 2D), confirming their suitability for further investigation. Employing stringent screening criteria (*P*-value < 0.05, VIP > 1, and fold change > 1.5), 162 differentially expressed metabolites were identified, including 84 significantly upregulated and 78 significantly downregulated species. These metabolites were further ranked based on log_2_(fold change) magnitude and visualized in a scatter plot (Fig. 2E).

The thawed group exhibited a significant increase in multiple lyso-phospholipids (Lyso-PLs) compared to the fresh group (Fig. 2E), suggesting a potential association with ferroptosis [40, 43].

### Impact of ferroptosis and apoptosis inhibitors on sperm motility assessment

Inhibitor rescue serves as a primary method for discerning the type of cell death. Optimal concentrations of ferroptosis inhibitors, including 2 μmol/L DFO, 2 μmol/L Fer-1, and 5 μmol/L Lip-1, significantly improved sperm TM compared to control groups C1 and C2 (Table 2). Although the optimal concentration (5 μmol/L) of the apoptosis inhibitor Z-VAD showed some improvement relative to control groups C1 and C2, the difference was not statistically significant (Table 2). These inhibitors at optimized concentrations were used for subsequent experiments; these findings suggest the involvement of both ferroptosis and apoptosis in RCD is associated with sperm freezing damage, with ferroptosis potentially playing a more dominant role.

**Table 2:** Effects of regulated cell death (RCD) inhibitors on the total motility (TM) and progressive motility (PM) integrity of frozen-thawed goat sperm

GROUP	TM, %	PM, %	VAP, μm/s	VSL, μm/s	VCL, μm/s	STR, %	LIN, %	ALH, μm	BCF, Hz
C1	32.60 ± 2.29^f^	17.40 ± 2.91^de^	20.70 ± 2.64^g^	18.22 ± 3.36^fg^	33.09 ± 5.91^f^	27.27 ± 2.97^f^	19.00 ± 2.25^f^	2.12 ± 0.47^efgh^	6.54 ± 2.53^d^
C2	34.46 ± 2.85^f^	23.10 ± 1.98^cd^	23.30 ± 1.69^efg^	19.94 ± 1.42^efg^	36.16 ± 2.29^ef^	29.61 ± 2.89^ef^	19.62 ± 2.09^f^	1.94 ± 0.11^fgh^	8.16 ± 0.78^cd^
DFO (2 μmol/L)	46.08 ± 1.62^bcd^	24.10 ± 4.66^c^	26.86 ± 2.84^def^	22.94 ± 2.19^cdef^	40.65 ± 5.02^def^	39.5 ± 1.88^bcd^	26.97 ± 1.87^bcd^	2.47 ± 0.13^cdef^	10.83 ± 0.57^ab^
DFO (5 μmol/L)	42.53 ± 3.55^de^	25.30 ± 1.42^bc^	29.56 ± 0.99^bcd^	25.04 ± 0.88^bcd^	43.39 ± 2.47^cde^	35.82 ± 4.37^cde^	25.23 ± 3.18^cde^	2.59 ± 0.04^bcde^	9.08 ± 1.47^bc^
DFO (10 μmol/L)	37.45 ± 6.67^ef^	16.03 ± 8.74^e^	20.81 ± 9.31^g^	17.93 ± 7.39^g^	30.99 ± 15.36^f^	32.63 ± 5.40^ef^	25.14 ± 4.26^cde^	1.81 ± 0.84^gh^	9.32 ± 2.92^bc^
Fer-1 (2 μmol/L)	50.16 ± 4.39^abc^	31.66 ± 2.10^ab^	37.02 ± 2.38^a^	31.63 ± 2.06^a^	53.98 ± 2.53^ab^	42.92 ± 4.88^ab^	29.92 ± 3.85^abc^	3.02 ± 0.26^bc^	10.53 ± 1.28^ab^
Fer-1 (5 μmol/L)	43.03 ± 4.69^cde^	27.70 ± 1.63^abc^	33.59 ± 3.71^abc^	28.16 ± 2.54^ab^	48.80 ± 6.24^bcd^	35.88 ± 3.90^cde^	25.34 ± 2.48^cde^	2.91 ± 0.43^bcd^	8.09 ± 1.44^cd^
Fer-1 (10 μmol/L)	38.63 ± 1.68^def^	24.30 ± 3.12^c^	28.10 ± 2.57^cde^	23.18 ± 1.72^cde^	43.60 ± 3.22^cde^	32.04 ± 1.59^ef^	21.39 ± 1.56^ef^	2.64 ± 0.28^bcde^	8.36 ± 0.63^cd^
Lip-1 (2 μmol/L)	51.46 ± 2.61^ab^	25.48 ± 2.34^bc^	35.34 ± 1.42^ab^	28.09 ± 1.32^ab^	61.17 ± 2.69^a^	41.34 ± 2.39^abc^	24.83 ± 1.19^de^	3.65 ± 0.22^a^	12.11 ± 0.77^a^
Lip-1 (5 μmol/L)	54.15 ± 5.81^a^	33.38 ± 3.49^a^	31.83 ± 3.58^abcd^	27.32 ± 3.15^abc^	47.28 ± 6.54^bcd^	46.79 ± 5.92^a^	32.59 ± 3.57^a^	2.82 ± 0.47^bcd^	10.58 ± 0.64^ab^
Lip-1 (10 μmol/L)	53.58 ± 7.33^ab^	31.10 ± 3.48^ab^	33.27 ± 4.12^abc^	28.02 ± 3.66^ab^	50.97 ± 4.64^bc^	45.27 ± 6.49^ab^	30.48 ± 4.98^ab^	3.21 ± 0.47^ab^	11.31 ± 1.52^ab^
Z-VAD (2 μmol/L)	39.30 ± 3.48^def^	25.48 ± 4.19^bc^	26.09 ± 4.91^defg^	22.68 ± 3.09^cdefg^	39.97 ± 8.41^def^	33.26 ± 2.89^def^	23.07 ± 3.23^def^	2.35 ± 0.63^defg^	7.85 ± 0.87^cd^
Z-VAD (5 μmol/L)	40.10 ± 4.00^def^	25.80 ± 2.76^bc^	26.55 ± 2.94^defg^	22.14 ± 2.54^defg^	40.33 ± 3.90^def^	33.6 ± 4.07^def^	22.79 ± 3.46^def^	2.42 ± 0.24^cdefg^	8.07 ± 0.56^cd^
Z-VAD (10 μmol/L)	34.38 ± 5.43^f^	21.40 ± 2.60^cde^	21.03 ± 2.04^fg^	18.31 ± 2.07^fg^	31.06 ± 3.04^f^	29.87 ± 6.06^ef^	20.92 ± 4.43^ef^	1.69 ± 0.19^h^	7.87 ± 1.83^cd^

*TM* Total motility, *PM* Progressive motility, *VAP* Average path velocity, *VSL* linear motion velocity, *VCL* Curvilinear motion velocity, *STR* Straightness, *LIN* Linearity, *ALH* Amplitude of lateral head displacement, *BCF* beat cross frequency

^a–h^In the same column, different letters indicate significant differences, with *P* < 0.05. The presence of the same letter indicates non - significant differences, with *P* = 0.05

### Effect of ferroptosis and apoptosis inhibitors on sperm plasma membrane and acrosome integrity and RCD proportions

**Fig. 3:**
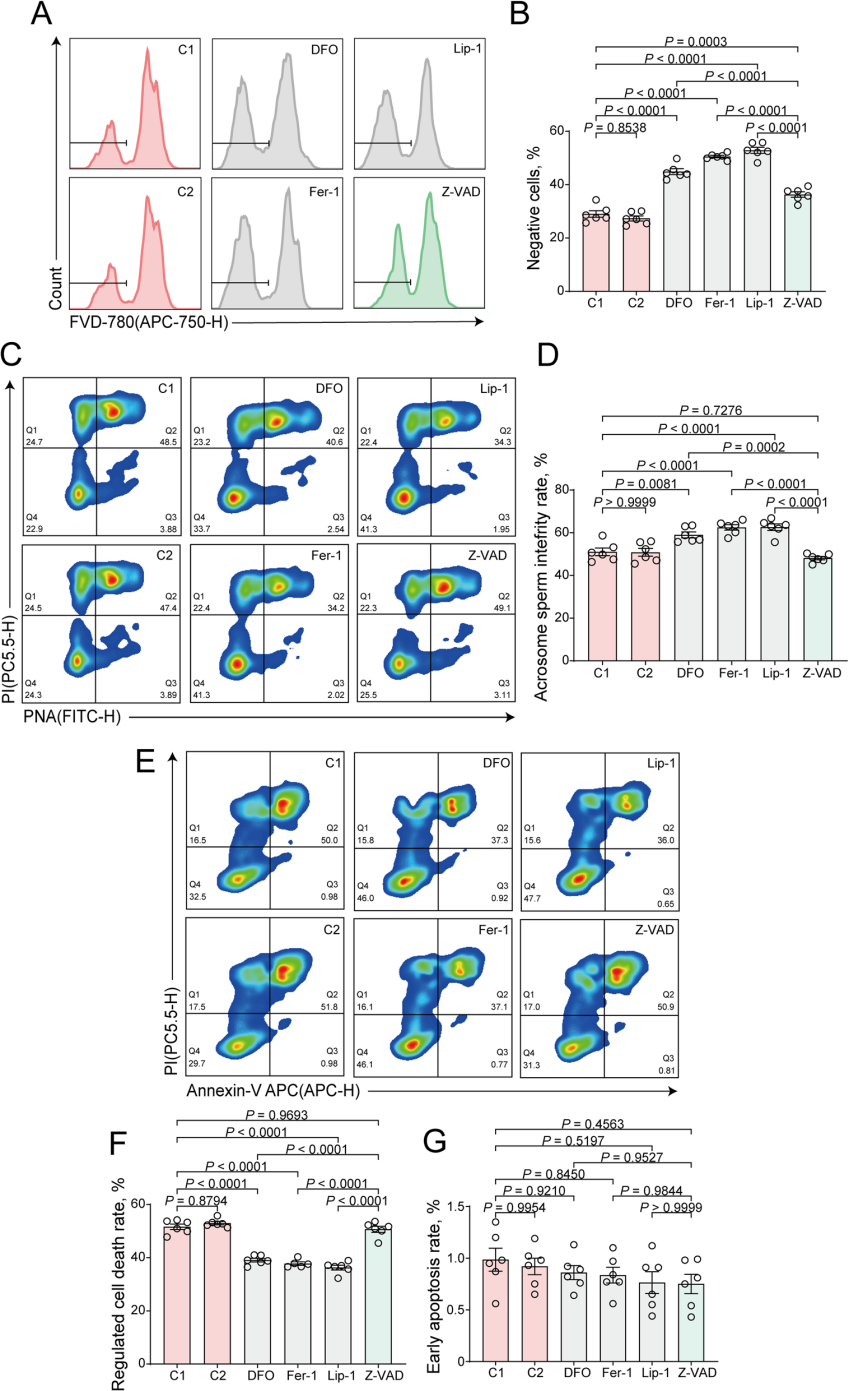
Effects of ferroptosis and apoptosis inhibitors on sperm plasma membrane and acrosome integrity and RCD proportions of frozen-thawed goat sperm. **A** Flow cytometric analysis of plasma membrane integrity of frozen-thawed sperm treated with RCD inhibitors prior to freezing, with representative data from each group shown. The gated portion represents cells with intact plasma membranes. **B** Statistical graph of plasma membrane integrity for each experimental group in panel A. **C** Flow cytometric analysis of acrosome integrity of frozen-thawed sperm treated with RCD inhibitors, with representative data for each group shown. Quadrant analysis: Q1 represents cells with intact acrosomes and damaged plasma membranes (FITC − , PI +), Q2 represents cells with damaged acrosomes and damaged plasma membranes (FITC + , PI +), Q3 represents apoptotic cells with intact plasma membranes (FITC + , PI −), and Q4 represents cells with intact acrosomes and intact plasma membranes (FITC − , PI −). **D** Statistical graph of Q1 + Q4 for each experimental group in panel C. **E** Flow cytometric results show the occurrence of RCD in frozen-thawed sperm treated with RCD inhibitors, with representative data for each group. Quadrant analysis: Q1 may represent cell debris or cells undergoing apoptotic cell death (APC − , PI +), Q2 represents cells undergoing RCD (APC + , PI +), Q3 represents early apoptotic cells (APC + , PI −), and Q4 represents live cells (APC − , PI −). **F** Statistical graph of Q2 + Q3 for each experimental group in panel E, indicating the overall occurrence of RCD. **G** Statistical graph of Q3 for each experimental group in panel E, representing the early apoptotic cell population

To assess sperm freezing damage, we investigated the integrity of sperm plasma membranes. Across the experimental groups, we observed a significantly higher percentage of intact plasma membranes in all RCD inhibitor groups compared to the control groups (Fig. 3A and B). Notably, the apoptosis inhibitor group showed significantly lower membrane integrity levels compared to the ferroptosis inhibitor groups (Fig. 3A and B).

In addition to plasma membrane integrity, we evaluated the integrity of sperm acrosomes. Our analysis revealed a significantly higher percentage of intact acrosomes in the ferroptosis inhibitor groups compared to both the control and apoptosis inhibitor groups (Fig. 3C and D). Conversely, no significant difference in acrosome integrity was observed between the apoptosis inhibitor group and the control group (Fig. 3C and D). Taken together, these findings underscore the protective role of ferroptosis inhibitors in preserving sperm membrane and acrosome integrity, highlighting their potential significance in mitigating cryopreservation-induced damage.

Annexin V/PI double staining, a widely used method for detecting apoptosis, can also identify non-apoptotic RCD. Early apoptotic cells are typically used as apoptotic markers when multiple forms of RCD are present [44–47]. Here, cells undergoing RCD were categorized into Annexin V + /PI + and Annexin V + /PI − populations, with the latter representing early apoptotic cells. The proportion of cells undergoing RCD was significantly lower in the ferroptosis inhibitor groups compared to both the control and apoptosis inhibitor groups (Fig. 3E and F), while no significant difference was observed between the apoptosis inhibitor and control groups (Fig. 3E and F). Notably, the population of early apoptotic cells remained consistently low (approximately 1%) across all groups (Fig. 3E and G).

### Effects of ferroptosis and apoptosis inhibitors on sperm ROS levels, lipid peroxidation, and Fe^2+^ levels

**Fig. 4:**
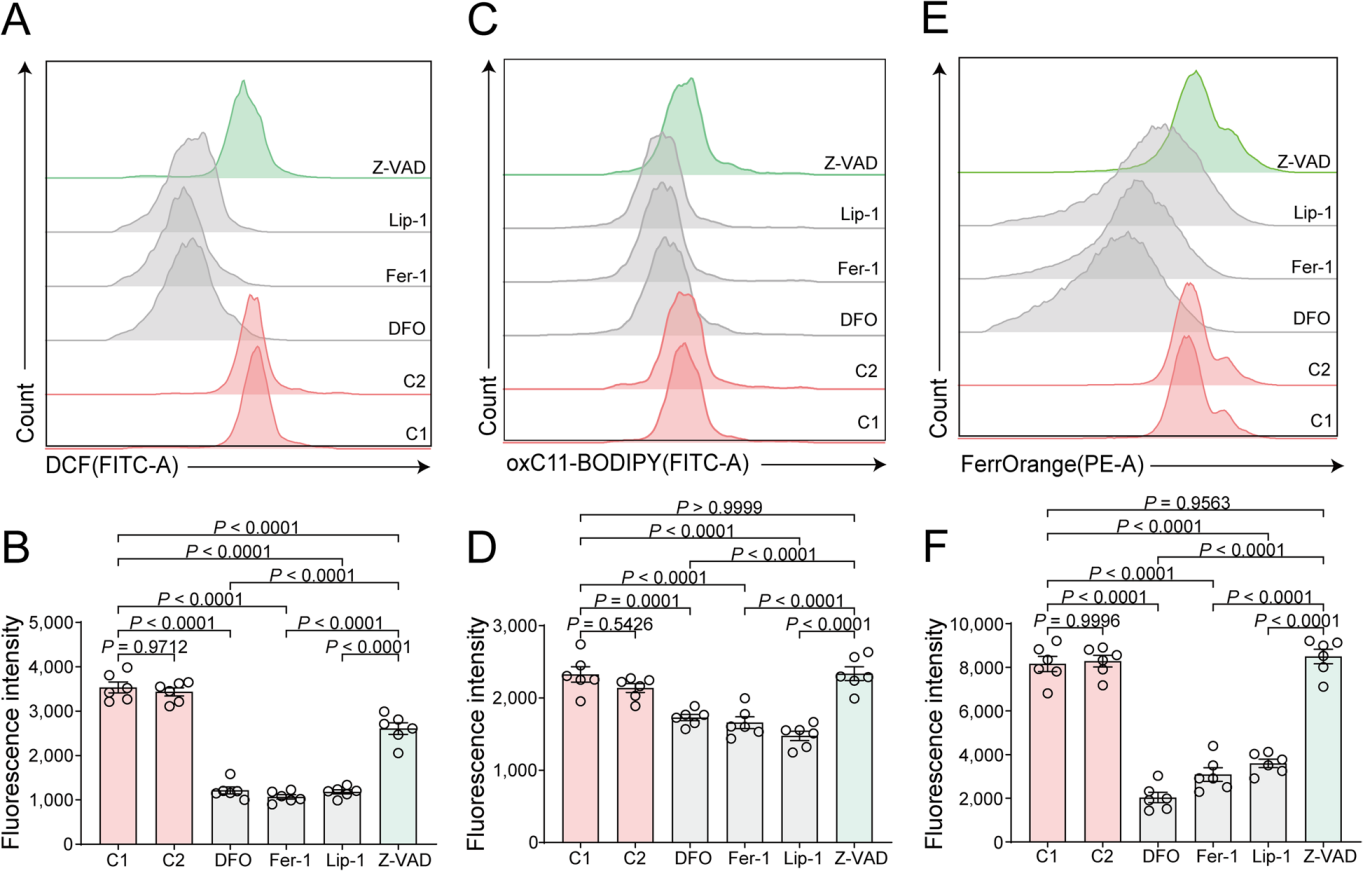
Effects of different regulated cell death (RCD) inhibitors on the ROS levels, lipid peroxidation, and Fe^2+^ levels of frozen-thawed goat sperm. **A** Flow cytometric results for ROS detection in frozen-thawed sperm treated with RCD inhibitors, displaying representative data for each group. **B** Statistical graph of the average dichlorofluorescein (DCF) fluorescence intensity for each experimental group in panel A, indicating ROS levels. **C** Flow cytometric results for lipid peroxidation detection in frozen-thawed sperm treated with RCD inhibitors, displaying representative data for each group. **D** Statistical graph of the average oxC11-BODIPY fluorescence intensity for each experimental group in panel C, representing lipid peroxidation levels. **E** Flow cytometric results of Fe^2+^ levels in frozen-thawed sperm treated with RCD inhibitors, with representative data displayed for each group. **F** Statistical graph of the average FerrOrange fluorescence intensity, representing Fe^2+^ levels, for each experimental group in panel E

ROS can trigger both apoptosis and ferroptosis, with ROS levels positively correlated with sperm freezing damage. Our analysis of ROS levels showed that all RCD inhibitors significantly decreased intracellular ROS levels compared to the control groups (Fig. 4A), with the ferroptosis inhibitor groups displaying significantly lower levels than the apoptosis inhibitor group (Fig. 4B). Excessive membrane lipid peroxidation serves as a specific marker of ferroptosis.

Lipid peroxidation results demonstrated significantly lower levels in the ferroptosis inhibitor groups compared to both the control and apoptosis inhibitor groups (Fig. 4C), while no significant differences were observed between the apoptosis inhibitor group and the control group (Fig. 4D).

The results further revealed significantly decreased Fe^2+^ levels in the ferroptosis inhibitor groups compared with both the control group and the apoptosis inhibitor groups (Fig. 4E). No statistically significant differences in Fe^2+^ levels were noted between the apoptosis inhibitor groups and the control group (Fig. 4F). These results underscore the potency of ferroptosis inhibitors in mitigating sperm freezing damage, as evidenced by their ability to significantly reduce ROS levels, lipid peroxidation, and Fe^2+^ levels compared with apoptosis inhibitors.

### Analysis of marker protein expression reveals ferroptosis as the dominant mechanism in sperm freezing damage

**Fig. 5:**
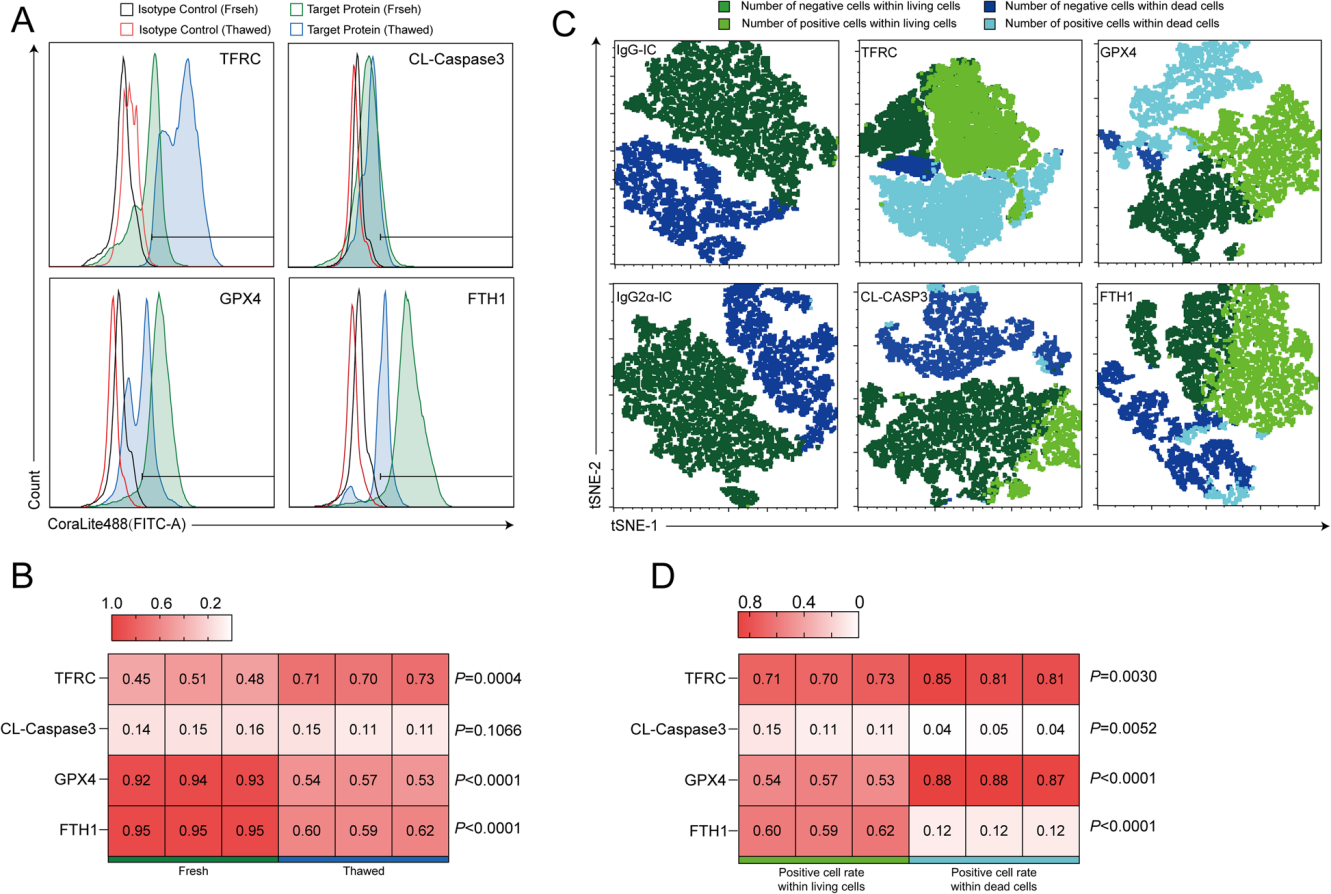
Detection of expression levels of key proteins involved in ferroptosis and apoptosis by flow cytometry. **A** Expression levels of various proteins in fresh live sperm and frozen-thawed live sperm. **B** Heatmap illustrating the expression data from panel A. **C** Expression levels of various proteins in dead and live cells of frozen-thawed sperm. **D** Heatmap illustrating the expression data from panel C

Expression levels of these markers were assessed using specific isotype controls to ensure staining specificity (Fig. 5A and B). CoraLite488-positive signals of isotype controls in all groups were consistently below 1%, indicating minimal non-specific staining and accurate gating.

The positive rate of TFRC in live cell populations of fresh sperm (47.8%) was significantly lower than that in live cell populations of frozen-thawed sperm (71.5%) (Fig. 5A and B), and the positive rate of TFRC in live cell populations of frozen-thawed sperm (71.5%) was significantly lower than that in dead cell populations (82.5%) (Fig. 5C and D).

Conversely, there was no significant difference in the positive rate of CL-caspase3 between live cell populations of fresh sperm (14.6%) and frozen-thawed sperm (11.9%) (Fig. 5A and B); however, the positive rate of CL-caspase3 in live cell populations of frozen-thawed sperm (11.9%) was significantly lower than that in dead cell populations (4.3%) (Fig. 5C and D). The trend of TFRC expression changes suggests ferroptosis as the predominant form of RCD in sperm freezing damage.

The positive rate of GPX4 in viable cell populations of fresh sperm (92.9%) was significantly higher than that in viable cell populations of frozen-thawed sperm (54.5%) (Fig. 5A and B). Interestingly, the positive rate of GPX4 in viable cell populations of frozen-thawed sperm (54.5%) was significantly lower than that in dead cell populations (87.8%) (Fig. 5C and D). Consequently, we speculate that the occurrence of ferroptosis during sperm cryopreservation is not primarily caused by the degradation of GPX4 but rather indicates a potential compensatory mechanism employed by sperm to counteract ferroptosis.

Intriguingly, the positivity rate of FTH1 in live cell populations of fresh sperm (94.8%) was significantly higher than that in live cell populations of frozen-thawed sperm (60.4%) (Fig. 5A and B), whereas the positivity rate of FTH1 in live cell populations of frozen-thawed sperm (60.4%) was significantly higher than that in dead cell populations (11.9%) (Fig. 5C and D), contrasting with the trend observed for TFRC.

## Discussion

In the context of sperm freezing damage, a key area of investigation lies in elucidating the mechanisms underlying RCD, particularly those induced by oxidative stress. Oxidative stress, a significant contributor to sperm freezing damage, triggers various forms of RCD, notably ferroptosis and apoptosis [12]. These processes are characterized by distinct biochemical pathways that lead to cell demise [17]. Identifying the predominant form of RCD operative during sperm cryopreservation is crucial as this information can inform the development of targeted interventions to improve the survival and quality of frozen-thawed sperm. Therefore, a comprehensive understanding of oxidative stress-induced RCD mechanisms, including but not limited to ferroptosis- and apoptosis-related pathways, is imperative for enhancing the outcomes of sperm cryopreservation protocols.

In our study, ferroptosis was identified as the primary form of RCD responsible for goat sperm freezing damage, superseding apoptosis. Although ferroptosis has not been previously investigated in the context of sperm freezing damage, numerous studies have hinted at its importance in this regard [12].

Our findings indicated a significant increase in Lyso-PL after sperm cryopreservation. We hypothesize that this increase is related to the involvement of ferroptosis. Specifically, ferroptosis involves ROS-mediated oxidation of PUFA-PL into PUFA-PL-OOH, which is subsequently cleaved by iPLA2 into Lyso-PL and oxidized PUFA. The accumulation of Lyso-PL during this self-perpetuating process underscores the potential role of ferroptosis in sperm freezing damage [21].

The elevated PUFA content in the plasma membrane, a hallmark of sperm maturation [23–27], further supports this hypothesis. Considering the relationship between ferroptosis and PUFA-PL oxidation, coupled with sperm's inherently limited antioxidant capacity, oxidative stress-induced ferroptosis appears inevitable under the environmental stresses (e.g., low temperatures and osmotic pressures) encountered during cryopreservation. Therefore, we speculate that the increase in Lyso-PL after sperm cryopreservation constitutes an indicator of ferroptosis-mediated damage.

Primarily lipophilic antioxidants serve as ferroptosis inhibitors, including Lip-1 and Fer-1, which act by reducing PUFA-PL-OOH formation [48]. Our findings indicate that the ferroptosis inhibitors Lip-1 and Fer-1 significantly enhanced various sperm qualities compared with the apoptosis inhibitor Z-VAD. Specifically, these inhibitors improved sperm motion parameters, including TM and PM, as well as sperm quality parameters, such as sperm plasma membrane and acrosome integrity and RCD proportions. Furthermore, they effectively reduced ferroptosis markers, including ROS levels, lipid peroxidation, and Fe^2+^ levels, thereby mitigating oxidative damage during sperm cryopreservation. Although Z-VAD exhibited some improvement in certain quality parameters compared to the control group, it is evident that apoptosis is not the primary type of RCD involved.

Similar to Lip-1 and Fer-1, α-tocopherol (α-TOH) and Trolox are well-known ferroptosis inhibitors capable of reducing lipid peroxides [48]. These compounds have demonstrated promising effects on sperm cryopreservation. α-TOH enhances biochemical and motility parameters of cryopreserved boar semen [49]. Additionally, the addition of α-TOH to the diluent effectively reduced the degree of lipid peroxidation in horse sperm under low-temperature conditions (5 °C), thereby maintaining its motility [50]. Observations of the ultrastructure of cryopreserved goat semen revealed that Trolox preserved the integrity of the sperm plasma membrane and mitochondrial structures [51]. Furthermore, Trolox can safeguard the quality of cryopreserved semen from both normal individuals and patients with oligozoospermia [52].

The oxidation of PUFA-PL relies on iron-mediated Fenton reactions. Therefore, iron chelators such as DFO effectively reduce ferroptosis occurrence [48]. Studies involving induced oxidative stress models in sperm have shown DFO to rescue sperm from reduced motility and elevated lipid peroxidation levels [53, 54]. Moreover, simultaneous treatment with Trolox and DFO significantly improves sperm motility parameters and reduces oxidative stress by up to 20% [55].

The rescue effect of inhibitors frequently provides insights into the primary form of RCD in cells. However, it is essential to recognize that certain inhibitors might inadvertently induce alternative forms of RCD, underscoring the necessity of identifying key marker proteins. Consequently, elucidating the mechanisms governing ferroptosis in sperm during cryopreservation not only enhances our understanding of this process but also offers valuable insights into potential interventions aimed at mitigating sperm freezing damage.

Redox and iron regulation form the central framework of ferroptosis, with solute carrier family 7 member 11 (SLC7A11)-GSH-GPX4 constituting the main defense against its redox aspects [21]. Our study observed a high concordance in the expression levels of GPX4 and TFRC in both viable and non-viable sperm, suggesting that ferroptosis in frozen-thawed goat sperm is not solely caused by decreased GPX4 expression. This finding underscores the multifaceted nature of ferroptosis and its implications for sperm cryopreservation outcomes.

Meseguer et al. [56] found that the expression level of GPX4 in fresh sperm can predict the cryopreservation resistance of human sperm, underscoring the clinical relevance of our observations. Therefore, the inactivation of GPX4 during sperm cryopreservation could exacerbate sperm freezing damage. Notably, while the inhibition or degradation of GPX4 is sufficient for ferroptosis, the depletion of GSH alone can also induce it. As the content of GSH decreases during sperm cryopreservation [57, 58], sperm becomes more susceptible to ferroptosis, leading to cryoinjury. However, exogenous supplementation of GSH presents a potential therapeutic avenue for improving cryopreservation outcomes [59].

The synthesis of GSH in sperm may rely on the transport of cystine by SLC7A11, rather than the methionine cycle. Inhibition of SLC7A11 by the ferroptosis inducer sulfasalazine (SS) can result in decreased motility of frozen-thawed sperm and a reduced ability to bind to the zona pellucida [60, 61]. However, its role in goat sperm freezing damage is not yet clear.

The driving force behind lipid peroxidation involves enzymatic and non-enzymatic pathways, both associated with Fe^2+^ [62, 63].

Additionally, our study demonstrated the protective effects of DFO on frozen-thawed sperm and highlighted the pivotal role of iron-related proteins TFRC and FTH1 in sperm cryopreservation injury. The proportion of live cells expressing TFRC significantly increased from fresh to frozen-thawed sperm; the highest expression was observed in dead cells. This upregulation suggests an association with sperm damage. Conversely, the FTH1 positivity rate considerably decreased from live fresh sperm to live frozen-thawed sperm; it was substantially lower in dead cells, indicating a protective function against oxidative stress and cell death during cryopreservation.

These findings emphasize the importance of maintaining iron homeostasis to mitigate sperm injury during freezing and thawing. Iron regulation may occur through two pathways: (1) extracellular Fe^3+^ binds to transferrin and enters the cell via TFRC; and (2) Fe^3+^ is reduced within the endosome and subsequently released into the cytoplasm as Fe^2+^, facilitated by proteins such as six-transmembrane epithelial antigen of the prostate 3, divalent metal transporter 1, or SLC39A14 [64]. The upregulation of TFRC expression may also result from transcripts carried by spermatozoa [65, 66].

Additionally, ferritin bound with Fe^3+^ forms autophagosomes under the influence of microtubule-associated protein light chain 3 and autophagy-related genes 5 and 7, which then fuse with lysosomes to produce autolysosomes. The degradation of these proteins releases Fe^3+^, and, with the assistance of specific metal reductases, increases the concentration of Fe^2+^, ultimately leading to ferroptosis [41, 42]. The export of ferritin may dictate a cell's resistance to ferroptosis. During the early stages of cellular responses to ferroptosis stress, Prommin2 can secrete ferritin outside the cell via exosomes [67]. This observation potentially explains why sperm with high freezability from dairy goats exhibit lower FTH1 expression levels compared to sperm with low freezability [68]. In summary, our findings underscore the critical role of iron regulation, particularly through TFRC and FTH1 expression, in determining the susceptibility of sperm to cryopreservation-induced injury.

In addition to widespread cell death, the presence of a specific proportion of sperm in hyperactivated or acrosome-reacted states among thawed sperm represents another key indicator of freezing-induced damage. This physiological shift depends on the sperm's ability to absorb extracellular Ca^2+^. Strategies such as depleting Ca^2+^ from the culture medium or supplementing the thawing solution with the Ca^2+^ chelator EGTA have been shown to enhance the fertilization potential of thawed sperm [69, 70].

These observations may be linked to ferroptosis onset [12]. Notably, an elevated influx of Ca^2+^, which triggers morphological alterations including osmotic stress and cell rupture, is a defining characteristic of ferroptosis [17]. Recent research has revealed that treating sperm with varying concentrations of the SLC7A11 inhibitor SS significantly affects their motility [61]. In fresh horse sperm, low concentrations of SS enhance motility, mimicking hyperactivation, whereas higher concentrations reduce motility. In cryopreserved sperm, motility decreases regardless of the inhibitor concentration. This observation suggests that certain phenotypic changes observed in cryopreserved sperm mimic the progression of ferroptosis, transitioning from normal sperm to viable sperm with increased intracellular Ca^2+^ flux (with manifestations such as hyperactivation and acrosome reaction), and ultimately leading to cell death without cell rupture [12].

## Conclusions

This investigation has provided important insights into the mechanisms underlying sperm freezing damage, identifying ferroptosis as the primary form of RCD. Using a multifaceted approach that incorporated metabolomics analysis, inhibitor studies, and assessments of key marker proteins, we elucidated potential pathways involved in ferroptosis during cryopreservation.

Our study demonstrated that specific ferroptosis inhibitors, such as Lip-1 and Fer-1, exhibit significant protection against sperm freezing damage. These findings underscore the therapeutic potential of these inhibitors in mitigating cryoinjury and suggest promising avenues for improving sperm cryopreservation outcomes.

Despite these promising findings, practical challenges remain in applying ferroptosis inhibitors to goat sperm cryopreservation. Notably, we have not yet assessed crucial endpoints such as the impact of these inhibitors on fertilization rates or overall reproductive outcomes. Further research is needed to refine these therapeutic interventions and confirm their effectiveness in practical applications.

In summary, this study has advanced our understanding of the role of ferroptosis in goat sperm freezing damage and identified potential inhibitors to mitigate this damage. However, additional research is required to address practical challenges and evaluate the broader impact of these inhibitors on reproductive outcomes.

## Supplementary Information

Additional file1. The gating strategy for analyzing the results of flow cytometry in this research section.

## Data Availability

The original contributions presented in this study are included in the article, and further inquiries can be brought to the corresponding author.

## Abbreviations

α-TOH: α-Tocopherol
CL-caspase3: Cleaved caspase-3
DFO: Deferoxamine
DMSO: Dimethyl sulfoxide
Fer-1: Ferrostatin-1
FTH1: Ferritin heavy chain
GPX4: Glutathione peroxidase 4
GSH: Glutathione
Lip-1: Liproxstatin-1
Lyso-PLs: Lyso-phospholipids
NC-BWW medium: Non-capacitating sperm BWW culture medium
OPLS-DA: Orthogonal partial least squares discriminant analysis
PBS: Phosphate-buffered saline
PUFA: Polyunsaturated fatty acid
PUFA-PL: PUFA-phospholipid
RCD: Regulated cell death
ROS: Reactive oxygen species
SLC7A11: Solute carrier family 7 member 11
TFRC: Transferrin receptor
tSNE: T-distributed stochastic neighbor embedding
TM: Total motility
PM: Progressive motility
Z-VAD: Z-VAD-FMK
