# Supplementary material for: Ferroptosis emerges as the predominant form of regulated cell death in goat sperm cryopreservation
Source: J Anim Sci Biotechnol. 2025 Feb 18;16:26. doi: 10.1186/s40104-025-01158-0 (PMC11834235; doi:10.1186/s40104-025-01158-0)

The gating strategy for analyzing the results of flow cytometry in this research section.

Plasma membrane permeability


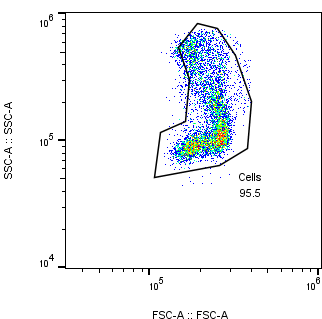

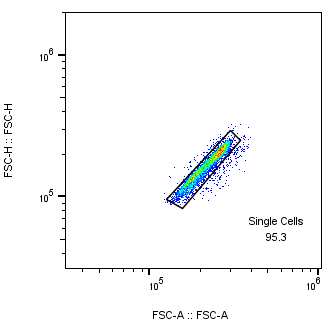

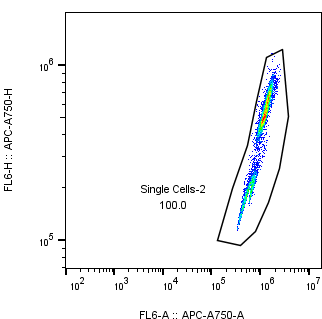

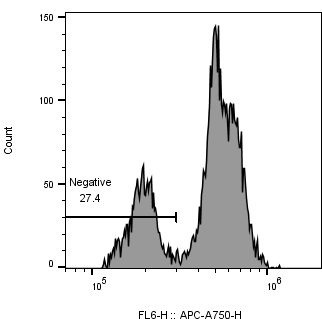


Assessment of acrosomal integrity


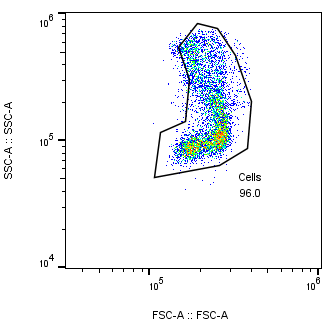

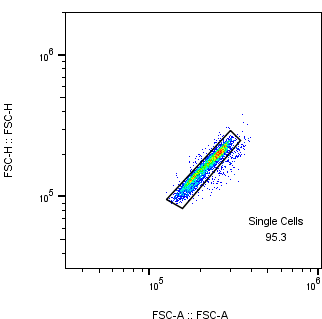

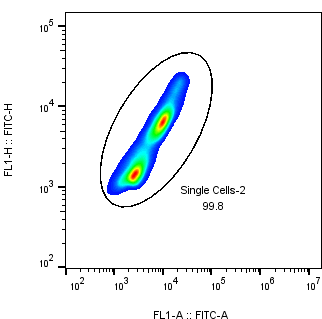

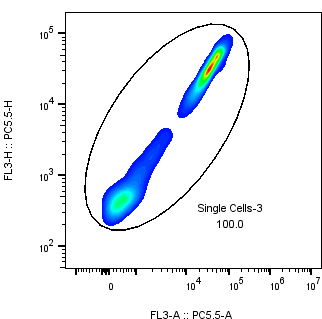

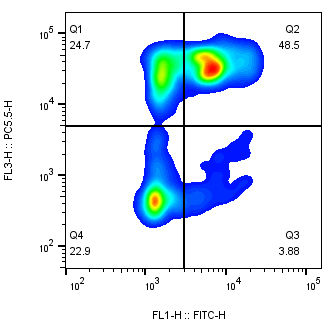


RCD Rate


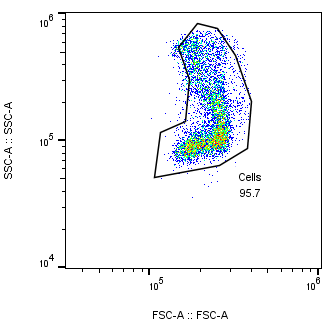

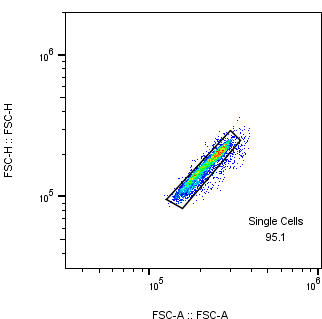

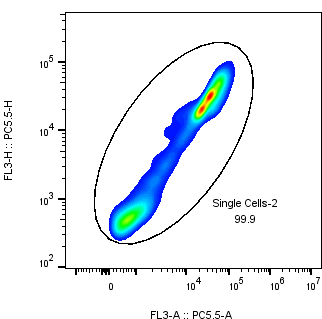

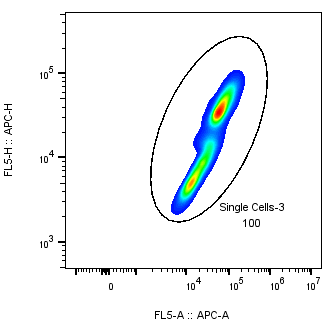

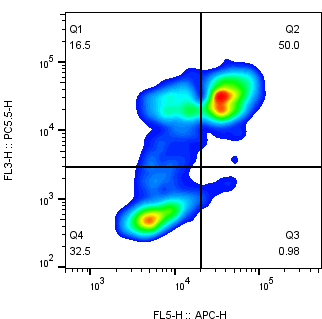


ROS


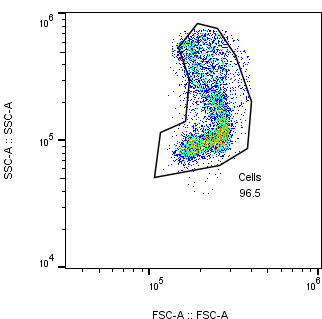

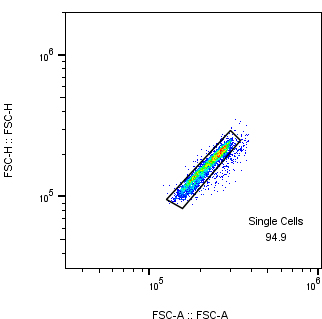

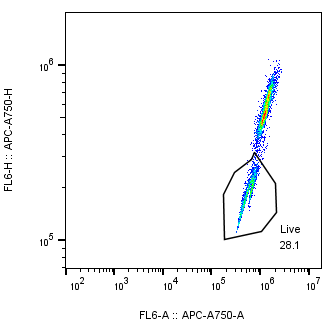

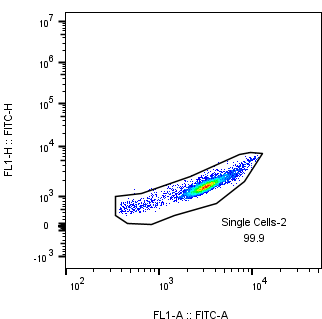

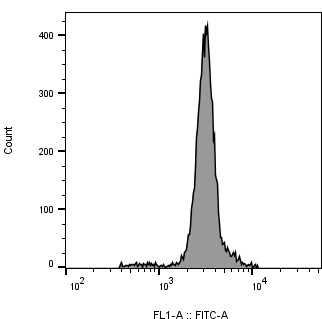


Lipid peroxidation


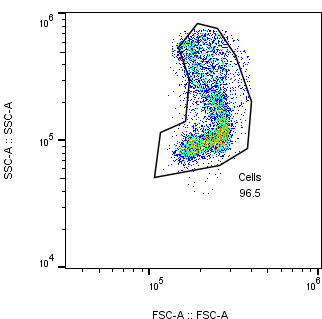

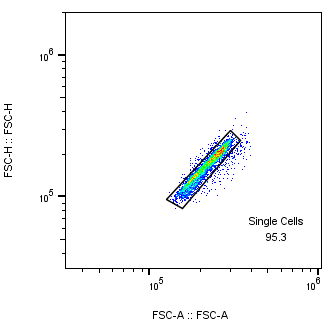

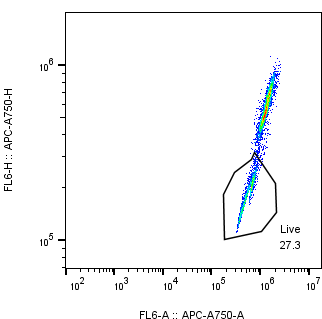

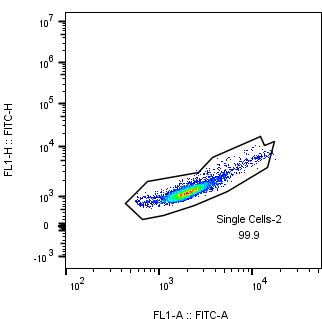

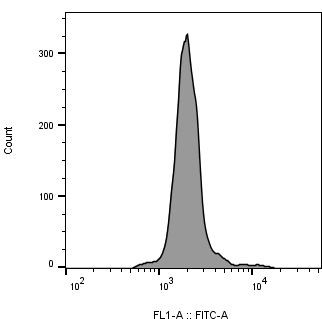


Fe^2+^


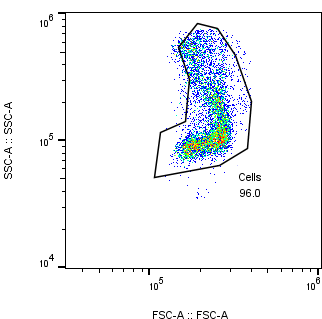

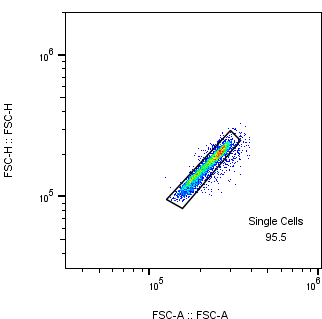

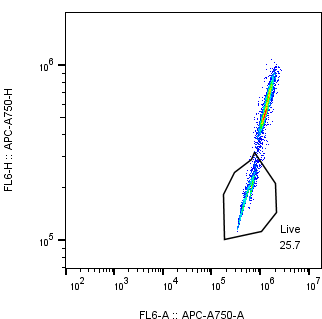

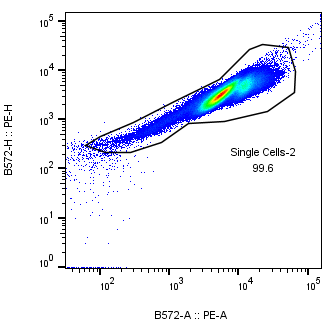

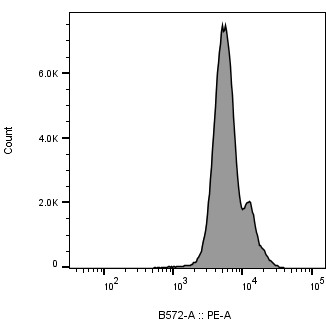

Supplement: Supplementary file 1 — Additional file1. The gating strategy for analyzing the results of flow cytometry in this research section. [file 40104_2025_1158_MOESM1_ESM.docx]
